# UpSMART: five years of digital innovation in cancer clinical research—achievements, challenges, and recommendations

**DOI:** 10.3389/fdgth.2025.1708067

**Published:** 2025-11-27

**Authors:** Paul O’Regan, Fouziah Butt, Louise Carter, Donna M. Graham, Anja Le Blanc, Richard Hoskins, Laura Stephenson, Akshita Patil, Muhammad Shabbir, Dilan Eken, Subir Singh, Andrea Villa, Luca Agnelli, Silvia Damian, Christopher Grave, Giulia Pretelli, Elena Garralda, Hannah Frost, Filippo de Braud, Andre Freitas, Caroline Dive, Harriet Unsworth

**Affiliations:** 1 Digital Cancer Research Team, CRUK National Biomarker Centre, The University of Manchester, Manchester, United Kingdom; 2 Division of Cancer Sciences, Faculty of Biology, Medicine and Health, The University of Manchester, Manchester, United Kingdom; 3 The Christie NHS Foundation Trust, Manchester, United Kingdom; 4 Research IT, The University of Manchester, Manchester, United Kingdom; 5 Department of Medical Oncology, Fondazione IRCCS Istituto Nazionale Tumori, Milan, Italy; 6 Department of Diagnostic Innovation, Fondazione IRCCS Istituto Nazionale Tumori, Milan, Italy; 7 Early Drug Development, Vall D'Hebron Institute of Oncology (VHIO), Barcelona, Spain; 8 Department of Medical Oncology, Vall D'Hebron University Hospital (HUVH), Barcelona, Spain; 9 Department of Hemato-Oncology, University of Milan, Milan, Italy; 10 Department of Computer Science, The University of Manchester, Manchester, United Kingdom; 11 IDIAP Research Institute, Martigny, Switzerland; 12 Cancer Research UK National Biomarker Centre, The University of Manchester, Manchester, United Kingdom

**Keywords:** digital healthcare products, cancer clinical trials, clinical trial innovation, digital health technologies, open-source

## Abstract

UpSMART, a research programme involving 24 European cancer centres, aimed to promote digital innovation in early-phase clinical research addressing challenges in recruitment, data collection and analysis. Several open-source digital healthcare products (DHPs) were developed through UpSMART, including eTARGET and trialFinder for trial matching, and PROACT 2.0 for patient-reported data. Lessons learned highlight the importance of multidisciplinary teams, sustainable funding and deployment, and engagement with the research community to maximise impact.

## 1 Introduction

The global cancer burden is growing—more than 35 million new cases are predicted in 2050, representing a 77% increase compared with 2022 (1). The number of cancer deaths per year is estimated to increase by almost 90% over this period (2). Clinical research in oncology will be crucial to try and mitigate these effects. The number of active cancer trials globally has increased more than 10-fold between 2000 and 2021 (3). However, this increase in trial activity creates a growing need to optimise trial delivery and analysis.

Digital technologies have the potential to improve all stages of the clinical trial process, including recruitment & retention, data collection and analytics (4,5). The benefits could include improved patient experience, cheaper, more efficient trials and more informed decision-making. Many digital tools have become available that could enable these benefits to be realised. However, barriers to the implementation of such technologies include regulatory requirements, data privacy concerns, and resource/infrastructure pressures (6,7).

In order to drive digital innovation in cancer clinical trials, we established UpSMART—a consortium of 24 cancer research centres across the United Kingdom, Italy, Spain and France (8,9). The UpSMART programme aimed to promote adoption of digital healthcare products (DHPs) in order to enhance digital capabilities and enable more informative, more efficient early-phase cancer clinical trials (8). In turn, this would improve the competitiveness of the UK and European clinical trials landscape to commercial trial sponsors and ultimately benefit patients by encouraging more early phase trials (8). We sought to create a repository of DHPs and methods that could be shared with the rest of the clinical trials community. DHPs would be freely distributed through open-source licensing, with the intention that the code could be further modified and improved by a community of users.

UpSMART has been delivered through a ‘hub and spoke’ model with an international coordinating centre based in Manchester and national coordinating centres in Italy and Spain. Together, these centres have managed activities across the other participating sites. The programme was initiated in February 2020 and is due to end in early 2026. Here, we present some of the tools and methods included under UpSMART, describe the challenges we encountered, and outline recommendations for future work to deliver digitised cancer clinical trials.

## 2 Achievements

**Figure 1:**
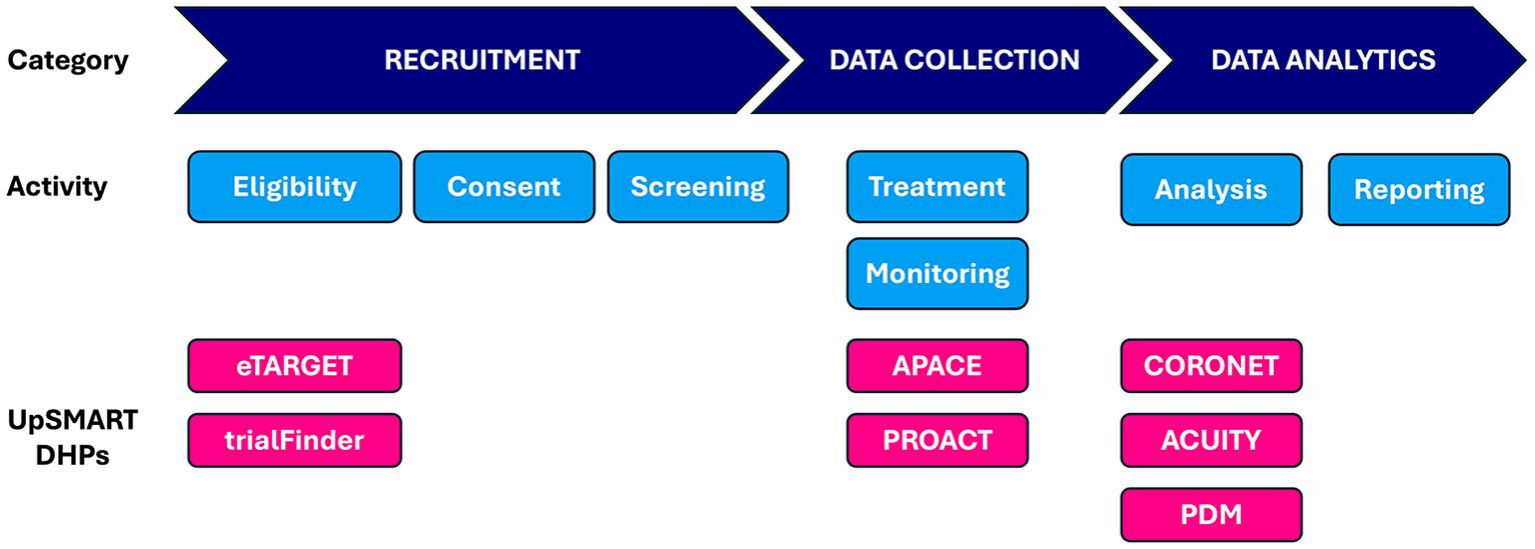
Elements of clinical trial process and corresponding UpSMART DHPs. APACE, Accelerometers to Measure Physical Activity in Cancer Patients on Early Phase Clinical Trials, CORONET, COVID-19 Risk in Oncology Evaluation Tool, DHP, Digital Healthcare Product; PDM: Protocol Deviation Monitoring tool; PROACT, Patient Reported Opinions About Clinical Tolerability.

Based on surveys conducted during the first year of UpSMART, 29 DHPs had been developed locally by participating centres, of which 8 were prioritised according to clinical need and the resource required for further development in order to release them as open-source products as part of UpSMART. We describe some of the DHPs successfully developed and released, which together address several of the key elements of the clinical trial process (Figure 1).

### 2.1 Digital tools to increase clinical trial recruitment

Although national guidelines strongly encourage participation in cancer clinical trials, enrolment rates remain low (10). One major challenge, particularly for precision oncology trials, is the recruitment and retention of participants (4). With up to 60% of cancer trials now requiring biomarker data for eligibility, next-generation sequencing (NGS) has become essential for identifying potential trials for patients (10). However, the synthesis and interpretation of patients’ molecular and clinical information can present a significant challenge to clinicians due to the volume and complexity of NGS data.

This challenge is exemplified by the TARGET trial, which aimed to match patients to clinical trials based on their tumour genome (measured in either tissue and/or blood) (11). Matching was carried out through a Molecular Tumour Board (MTB)—a multidisciplinary group of clinicians, geneticists and informaticians. However, the TARGET team identified a number of challenges affecting the efficient running of MTB meetings, notably the integration of patients’ clinical and molecular data (11). eTARGET—a web application that integrates patients clinical and genomic information—was developed to address these challenges. eTARGET offers several benefits, including: consolidation of clinical and genomic information into a single, searchable online platform; the ability for members to participate remotely; capture of meeting discussions; and automated generation of template results letters.

Access to matched treatment presents another challenge for MTBs, which are commonly employed in an advanced setting where no further standard of care treatment is available. Patients’ access to treatment may be dependent on a compassionate use or through a clinical trial (12). The lack of matched clinical trials is a common reason for the inability of patients to access treatment (12). The digital ECMT cancer trial matching tool (‘trialFinder’) was developed to support MTB members in the identification of matched trials (13). trialFinder is integrated with eTARGET and uses natural language processing to identify potential genomically matched trials for patients, then ranks the results according to biomarker enrichment and mechanistic reasoning.

Both eTARGET and trialFinder were co-developed with clinicians. They have been deployed to support the TARGET, CUP-COMP and TARGET-National trials, which have together recruited over 3,000 participants to date (11,14,15). The improved efficiency offered by eTARGET and trialFinder have been important factors in enabling these trials to operate at this scale. In addition, a public instance of the trialFinder has been deployed for more widespread use.

### 2.2 Digital health data collection

Traditional clinical data collection typically occurs during in-person visits, which can create logistical and financial challenges for patients, especially those with high morbidity (16). Furthermore, data collected at study visits represent only a snapshot of the patient’s status. DHPs have the potential to reduce the number of study visits, and can enable the collection of continuous or hard-to-obtain data, such as patient-reported outcomes and biomarkers based on wearables or mobile devices (16).

For example, PROACT is a DHP for communication between participants in early-phase cancer trials and their medical team (17). Participants can communicate via video, audio or text, providing *unstructured* feedback about their experiences on treatment (17). This type of information is particularly useful for early-phase studies when the potential toxicities of interventions are unknown. Pilot studies showed that PROACT provided a richer set of data that supplemented those data collected through conventional case report forms (17). However, the original version of PROACT included design features that made it difficult and costly to maintain over time, requiring ongoing updates and support that limited its wider usage.

PROACT 2.0, developed under the UpSMART programme, has been extensively refactored to address these limitations (18). PROACT 2.0 uses a simplified architecture to support easier deployment, and can collect both unsolicited (via text, audio or video) and solicited feedback (through customisable questionnaires). After a successful initial pilot study, PROACT 2.0 is currently being used in two further studies:

- The PROMOTE study, which monitors adverse events and quality of life in patients with metastatic colorectal cancer treated with anti-VEGF drugs (19)
- The Cancer Core Europe DART (CCE-DART) work package 12 sub-study, which aims to evaluate the feasibility of using digital tools to report effects of drugs in patients on phase 1 or 2 anticancer drug trials (20)

PROACT 2.0 has been released under open-source licence, and further development is planned to incorporate new functionality (21).

Another area of growing interest is the integration of continuous, real-world data streams, which could complement tools like PROACT and further enrich trial datasets. APACE is a multinational study delivered through UpSMART to evaluate the feasibility of collecting continuous physical activity and sleep data from patients with advanced cancer on early phase clinical trials (22). These data could lead to a better understanding of activity levels, sleep and fatigue experienced by participants on such trials, which could in turn help to improve interventions, management, and access to appropriate treatments. The trial aims to recruit 40 participants from 8 centres across three countries (UK, Spain and Italy), demonstrating the UpSMART programme's capability to conduct complex, multinational studies.

### 2.3 Digital analytics

Health data can be digitally collected on a large scale and in near real-time, offering significant opportunities to enhance healthcare through advanced analytics and clinical decision support. However, the scale of data can also present a challenge. For example, early-phase clinical trials require investigators to analyse and interpret large volumes of emerging data in order to inform decisions regarding dose escalation. Furthermore, investigators may want to explore whether patients’ biomarker status modifies the treatment effect (23).

One way in which DHPs can help address these challenges is by supporting investigators in the exploration of emerging trial data, in order to identify trends and develop hypotheses. ACUITY is a clinical dashboard that presents a set of data-driven interactive visualisations, at either the individual subject or population level. An early version of ACUITY was developed by AstraZeneca and after substantial refactoring and redevelopment through UpSMART, ACUITY has been released under open-source licence for use by the clinical research community (24, 25).

Digital technologies can further support clinical decision-making by identifying patterns within complex, high-dimensional datasets. Machine learning and artificial intelligence can be used to identify latent patterns across datasets such as these (26). The potential benefits of DHPs that use machine learning are illustrated by CORONET, a DHP developed under UpSMART during the COVID pandemic. It was known that cancer patients were at increased risk from COVID-19, but they presented with heterogeneous symptoms that were difficult to distinguish from the complications of cancer and its therapy. CORONET is designed to aid clinicians in deciding whether to admit cancer patients with symptoms of COVID-19 to hospital (27). CORONET uses a Random Forest model trained on real-world data to stratify patients according to their risk of severe complications. The clinical and laboratory tests used by CORONET are routinely available at hospital presentation, supporting its widespread use. The potential benefits are: optimisation of resources by targeting patients most likely to benefit from intensive monitoring, reduction in unnecessary hospitalisations leading to lower healthcare costs and reduced risk of infecting staff or other patients.

Finally, advances in language modelling can enable efficient processing of unstructured data at scale, which could generate novel insights. For example, information about protocol deviations is typically collected as free text, which makes aggregation and analysis within or across trials difficult. The Protocol Deviation Monitoring (PDM) tool, developed under UpSMART, uses advanced language modelling techniques to extract and structure data from protocol deviation reports, and provides an interface for researchers to visualise the results and look for patterns (28).

## 3 Challenges

UpSMART has delivered on its ambition to design, develop and release DHPs for cancer clinical trials. However, the team encountered a number of challenges during delivery of the programme. Here we describe some of these challenges, as they may be instructive for other teams aiming to introduce digital technologies into clinical trials.

First, availability of staff and infrastructure at research sites to deploy digital solutions. Information technology (IT) resources within hospital research facilities are often limited, and are understandably focussed on clinical care over research activity. We found that participating centres rarely had people with the time and skills needed to implement DHPs, which limited uptake. Infrastructure constraints can also present a barrier to the development and/or deployment of DHPs. For example, we found that limitations on compute resource available at collaborating hospitals hindered our capacity to train AI models locally.

Second, consideration of ethical, privacy, and regulatory factors is critical for the development of DHPs. Access to existing patient data on hospital infrastructure is rightly subject to rigorous data governance process, but this has been reported as a factor limiting the widespread use of real-world patient data (29). Collecting new types of data, or collecting data in new ways, presents additional challenges as there may be a lack of precedent, and governance boards may be reluctant to approve such approaches. For example, before the APACE study could start, hospitals required their own validation of the wearable devices and associated software. Obtaining the necessary approvals introduced delays in study startup, made more challenging due to the multinational nature of the study.

Third, the pace of development for artificial intelligence (AI) and other digital technologies is typically faster that than the pace of clinical evaluation in healthcare. This disparity could lead to challenges in integrating rapidly evolving AI tools into clinical practice, where thorough evaluation is essential to ensure safety and efficacy (30). Recent progress in the field of large language models (LLMs) illustrates the rapid pace of AI development—at the start of UpSMART, OpenAI's GPT-1 model had 117 m parameters and was limited to relatively simple tasks (31). In contrast, GPT-4 (published in 2023) had over 1 trillion parameters and was capable of complex tasks, including coding assistance, medical reasoning and multimodal AI (32). Whilst LLMs offer great potential to improve DHP performance, careful consideration of how to safely deploy them in the healthcare setting is required. For example, LLMs remain prone to ‘hallucinations’ –plausible-sounding content that is factually incorrect, unsupported by source data, or entirely fabricated (33). Furthermore, protection of patient data presents another challenge when using LLMs: either substantial local computational resources are needed to run models on-site, or patient data must be transmitted to cloud-based models, raising concerns about data privacy, security, and regulatory compliance (34).

## 4 Discussion

### 4.1 Recommendations

Based on our experience with UpSMART, we outline the following recommendations that could benefit future development and implementation of DHPs.

#### 4.1.1 Start with the clinical use case

Whilst digital skills are essential for the technical development and deployment of DHPs, input from clinicians and patients—who are typically the end-users—is vital to ensure that these tools address genuine clinical use cases and are compatible with existing healthcare workflows. (35) We recommend involving clinicians and/or patients from the earliest stages of development and throughout the implementation process. Their engagement ensures that DHPs are not only methodologically sound but also practically usable and relevant. Moreover, they can serve as effective champions among their peers, promoting uptake and use of DHPs. For example, clinical input and advocacy have been crucial in developing and promoting usage of eTARGET and trialFinder, and patients were consulted from the outset of PROACT 2.0 development to ensure the useability of the application.

#### 4.1.2 Invest in the digital workforce

Successful implementation of DHPs requires the right IT infrastructure, integration with existing IT systems, training for end users and continued support following deployment (36, 37). The UpSMART programme brought together a multidisciplinary central team that included oncologists, study managers, AI researchers and software engineers. The collaborative efforts of this team played a significant role in developing the chosen DHPs and making them available for use. However, recent studies report that although digital and analytics transformation are a high priority for healthcare organisations, most lack the necessary resources to implement these changes (38).

One way to address this could be to promote the hiring of dedicated digital teams at research sites. Funders could play a pivotal role in this regard, similar to their support for research nurses. There is some evidence from UpSMART that access to digital skills is improving—participating centres have reported that access to clinical informatician resource has become more common over the course of the programme. Nevertheless, assembling research teams that include people with digital skills is likely to be an important enabling factor for the wider implementation of DHPs.

#### 4.1.3 Provide software as a service (SaaS) to increase adoption

The goal of UpSMART was to release source code for DHPs, with the expectation that healthcare providers deploy and maintain the DHPs, ideally supported by a community of developers. However, the lack of dedicated digital teams available to perform these tasks limited the adoption and use of DHPs by participating centres, even where clinicians recognised the potential benefits.

Adoption of a SaaS model could mitigate this issue. SaaS delivered by a dedicated digital team from a central site could represent a more efficient allocation of resources compared with individual deployments at each site, by reducing duplication of roles and infrastructure. We recognise that SaaS puts the onus for support onto the hosting organisation and that funding such support is difficult within academia. Nevertheless, we expect that SaaS would substantially reduce barriers against adoption and increase clinical uptake of DHPs. To support SaaS provision, academic teams may need to explore alternative funding mechanisms, such as licensing agreements (e.g., royalties from commercial users to subsidise access for non-profit stakeholders) or partnerships with digital health companies that offer app-related services including technical support and maintenance.

#### 4.1.4 Engage with the research community

Having developed a DHP, it is important to engage with the research community in order to increase awareness and drive uptake. Without such efforts, the research community may remain unaware of available DHPs and their potential benefits, limiting their clinical impact. This is particularly important for products developed under the open-source model, which relies on collaboration between research groups to adapt and improve existing tools for the benefit of all.

In recognition of the importance of engaging with the research community, we convened a dedicated conference to present our findings. This event brought together a diverse audience, including healthcare providers, pharmaceutical companies, regulatory bodies, and patients. The event sparked substantial dialogue, and highlighted an interest in the development of digital healthcare products among the cancer research community.

## 5 Conclusions

The UpSMART programme has provided a rich set of DHPs that have been co-developed with patients and clinicians. The lessons learned from the programme and the recommendations outlined here provide guidelines for navigating complexity within the rapidly evolving field of digital healthcare, realising the potential offered by advances in technology, and ultimately improving patient outcomes.

## Data Availability

The original contributions presented in the study are included in the article/Supplementary Material, further inquiries can be directed to the corresponding author.
